# 3D Dynamic Culture of Rabbit Articular Chondrocytes Encapsulated in Alginate Gel Beads Using Spinner Flasks for Cartilage Tissue Regeneration

**DOI:** 10.1155/2014/539789

**Published:** 2014-11-24

**Authors:** Feiyue Xu, Lei Xu, Qi Wang, Zhaoyang Ye, Yan Zhou, Wen-Song Tan

**Affiliations:** State Key Laboratory of Bioreactor Engineering, School of Bioengineering, East China University of Science and Technology, 130 Mei-Long Road, P.O. Box 309, Shanghai 200237, China

## Abstract

Cell-based therapy using chondrocytes for cartilage repair suffers from chondrocyte dedifferentiation. In the present study, the effects of an integrated three-dimensional and dynamic culture on rabbit articular chondrocytes were investigated. Cells (passages 1 and 4) were encapsulated in alginate gel beads and cultured in spinner flasks in chondrogenic and chondrocyte growth media. Subcutaneous implantation of the cell-laden beads was performed to evaluate the ectopic chondrogenesis. It was found that cells remained viable after 35 days in the three-dimensional dynamic culture. Passage 1 cells demonstrated a proliferative growth in both media. Passage 4 cells showed a gradual reduction in DNA content in growth medium, which was attenuated in chondrogenic medium. Deposition of glycosaminoglycans (GAG) was found in all cultures. While passage 1 cells generally produced higher amounts of GAG than passage 4 cells, GAG/DNA became similar on day 35 for both cells in growth media. Interestingly, GAG/DNA in growth medium was greater than that in chondrogenic medium for both cells. Based on GAG quantification and gene expression analysis, encapsulated passage 1 cells cultured in growth medium displayed the best ectopic chondrogenesis. Taken together, the three-dimensional and dynamic culture for chondrocytes holds great potential in cartilage regeneration.

## 1. Introduction

Articular cartilage has a very poor self-repair capability. Currently established clinical treatments for cartilage damages often lead to inferior fibrocartilage-like reparative tissue compared to native hyaline cartilage. Autologous chondrocyte implantation (ACI), a cell-based strategy, holds great promise in regenerating cartilage defects [1]. In this approach, chondrocytes are acquired from non-load-bearing areas of articular cartilage, expanded* in vitro* and then injected into defects as a suspension [2]. Although several clinical studies have documented hyaline-like repair tissue and functional improvements with ACI, its long-term efficacy remains controversial [1, 3]. However, expansion of primary chondrocytes on two-dimensional (2D) plastic surfaces* in vitro* inevitably leads to the dedifferentiation [4]. Dedifferentiated chondrocytes display a fibroblast-like morphology, the reduced extracellular matrix (ECM) synthesis, downregulated chondrogenic gene expression (e.g.,* COL2A1*) and upregulated fibroblastic gene expression (e.g.,* COL1A1*), and an altered cell surface antigen profile [5]. Due to the loss of cartilage phenotype, dedifferentiated chondrocytes might play negative effects in cartilage regeneration [6].

Various strategies have been explored to achieve chondrocyte expansion while retaining cartilage phenotype. Barbero et al. applied a combination of platelet derived growth factor-BB (PDGF-BB), fibroblast growth factor-2 (FGF-2), and transforming growth factor-*β*1 (TGF-*β*1) to enhance cell proliferation [7]. Genetically modifying chondrocytes with exogenous genes of chondroinductive factors such as TGF-*β*1 and SOX-9 is promising but with controversy [4]. Considering the hypoxic microenvironment within articular cartilage, oxygen tension has also been manipulated during chondrocyte expansion [8, 9]. In addition, applying bioreactors such as spinner flasks in association with seeding of chondrocytes on microcarriers offers several advantages over conventional 2D culture including ease of scaling-up and mechanical stimulation [10, 11]. However, so far, the expanded chondrocytes in bioreactors do not always outperform those on 2D plastic surface, indicating that an optimized chondrocyte culture condition remains to be developed [9, 12].

3D condition is able to mimic naturally microenvironment of chondrocytes in cartilage tissue, which promotes cell-cell and cell-matrix interactions and enforce a rounded chondrocytic cell shape, thus maintaining their phenotype [5, 13]. Hydrogels made of agarose or alginate are highly hydrated 3D matrix and able to support the differentiation of freshly isolated chondrocytes in a long-term culture [14–16]. It has been documented that dedifferentiated chondrocytes encapsulated in alginate hydrogels can recover the chondrocytic phenotype [17–19]. On the other hand, it was reported that in response to the hydrostatic pressure, chondrocytes encapsulated in alginate gel beads secreted more glycosaminoglycans (GAG), a characteristic cartilaginous ECM component [20]. Additionally, fluid shear force in a spinner flask was implicated in stimulating ECM production by chondrocytes seeded in chitosan scaffolds [21]. Recently, Lee et al. found that culture of cellular aggregates of dedifferentiated chondrocytes in a spinner flask was able to induce the redifferentiation [22]. These studies suggest that mechanical stimulation of chondrocytes in 3D culture can be a promising strategy for maintaining the cartilage phenotype.

Hence, in the present study, it was hypothesized that 3D encapsulation in alginate gel beads in combination with the dynamic culture in a spinner flask could be beneficial in maintaining the phenotype of chondrocytes during* in vitro* culture. Rabbit articular chondrocytes (rACs) were encapsulated in alginate gel beads and cultured in spinner flasks for 35 d. In addition, two different passages of rACs (passage 1 (P1) and P4) and two different culture media (chondrogenic and growth media) were tested. Further, after the 35 d culture, cell-laden beads were subcutaneously implanted in nude mice to evaluate the ectopic chondrogenesis.

## 2. Materials and Methods

### 2.1. Isolation and Culture of Chondrocytes

All animal experiments were performed at Shanghai Laboratory Animal Center (SLAC, Shanghai) in accordance with the institutional guidelines of Animal Care and Use Committee of SLAC. rACs were isolated from 2-month-old New Zealand rabbits as previously described [23]. Articular cartilage tissue was finely minced, rinsed with phosphate-buffered saline (PBS), treated with 0.25% trypsin/EDTA (Gibco) for 30 min, and then digested with 0.1% collagenase type II (200 units/mg; Invitrogen) in serum-free high-glucose Dulbecco's modified Eagle's medium (DMEM; Gibco) for 6 h at 37°C. Cells were collected by centrifuging and resuspended in chondrocyte growth medium, which consisted of high-glucose DMEM, 10% fetal bovine serum (FBS; HyClone), 0.1 mM nonessential amino acids (Invitrogen), 0.4 mM proline (Invitrogen), 0.05 mg/mL vitamin C (Invitrogen), 100 units/mL penicillin (Invitrogen), and 100 units/mL streptomycin (Invitrogen). For expansion, rACs were plated at 1 × 10^4^ cells/cm^2^ and incubated in a humidified atmosphere of 5% CO_2_ at 37°C. Medium was refreshed every 2-3 d.

### 2.2. Chondrocyte Encapsulation and Culture in Spinner Flasks

Alginate solution was sterilized at 105°C for 20 min prior to encapsulation. rACs (P1 and P4) were harvested from culture, rinsed with 0.9% saline, and resuspended in 4% sodium alginate solution (low viscosity, Sigma) in 0.9% saline at a final cell density of 5 × 10^5^ cells/mL. The suspension was slowly dropped into 102 mM CaCl_2_ solution through a 25-gauged needle and incubated for 10 min to solidify gel beads, followed by rinse with 0.9% saline and DMEM sequentially.

In each spinner flask, 100 cell-laden alginate beads were loaded. The chondrogenic medium was composed of high-glucose DMEM, 10 mM NaHCO_3_, 10 mM N-(2-Hydroxyethyl)-piperazine-N′-(2-ethanesulfonic acid) (HEPES), 1% ITS + Premix (BD Biosciences), ascorbic acid (50 *μ*g/mL), 10 ng/mL TGF-*β*1 (PeproTech), 40 *μ*g/mL L-proline, 10^−7^ mol/L dexamethasone, 100 *μ*g/mL sodium pyruvate and 0.05 mg/mL vitamin C (Invitrogen), 100 units/mL penicillin (Invitrogen), and 100 units/mL streptomycin (Invitrogen). Both growth and chondrogenic media were applied for culture. Therefore, four different cultures were defined as (i) P1 rACs in chondrogenic medium (Ind-P1rAC); (ii) P1 rACs in growth medium (Con-P1rAC); (iii) P4 rACs in chondrogenic medium (Ind-P4rAC); and (iv) P4 rACs in growth medium (Con-P4rAC). All cultures were maintained at 50 rpm with 50 mL of medium. Medium was refreshed twice a week.

### 2.3. Scanning Electron Microscopy (SEM)

Cell-laden beads were rinsed with 0.9% saline, fixed in 4% paraformaldehyde at room temperature for 4 h, and then dehydrated in graded ethanol solutions followed by air drying. Samples were gold sputtered and examined using SEM (JSM-6360LV, JEOL) at 10 kV under high vacuum mode.

### 2.4. Live/Dead Staining

Cell viability was assessed using live/dead staining. Cell-laden beads were harvested, rinsed with 0.9% saline, and then incubated in 1 mL of DMEM containing 2 *μ*M calcein-AM (CAM; Sigma) and 2 *μ*M propidium iodide (PI; Sigma) at 37°C for 30 min. Fluorescent images were captured on a fluorescence microscope (Eclipse Ti-S, Nikon). Dead cells appear red and viable cells are green.

### 2.5. Biochemical Assays

To quantify DNA content and GAG, 3 beads were taken from each culture at different time points and treated with 300 *μ*L papain solution (125 *μ*g/mL papain (Sigma), 5 mM L-cysteine, 100 mM Na_2_HPO_4_, 5 mM EDTA, and pH 6.2) at 60°C overnight. DNA content was determined using Hoechst 33258 (Sigma) on Hoefer DQ300 fluorometer (Hoefer) as described [24]. Calf thymus DNA solution (Sigma) was used as a standard. GAG was measured following modified 1,9-dimethyl-methylene blue (DMMB)-based spectrometric method [25]. The papain-digested solution was added to DMMB solution (16 mg/L DMMB, 3.04 g/L glycine, and 2.37 g/L NaCl, pH 1.5) and the absorption at 525 nm was recorded on DU 730 UV/Vis spectrophotometer (Beckman Coulter). Chondroitin sulfate was used as a standard. Contents of DNA and GAG in one bead were presented. GAG content was also normalized to DNA content and expressed as GAG/DNA (*μ*g/*μ*g). Each test was performed in triplicate.

For MTT assay, at different time points, one bead was taken from each culture and treated with 240 *μ*L of 3-(4,5-dimethylthiazolyl-2)-2,5-diphenyltetrazolium bromide (MTT, 0.5 mg/mL; Sigma) in DMEM at 37°C for 4 h in dark. Insoluble formazan crystals reduced from MTT were extracted with DMSO. The absorbance (optical density (OD)) at 570 nm of the extractant was read on a microplate reader (ELx 800, Bio-Tek Instruments). Each test was performed in triplicate.

### 2.6. Histochemistry and Immunohistofluorescent Staining

Cell-laden beads were harvested on days 0 and 35, rinsed with 0.9% saline, fixed in 4% paraformaldehyde at room temperature for 4 h, embedded in OCT tissue freezing medium (Tissue-Tek), and cryosectioned into 10 *μ*m thick sections. Samples were stained with Safranin-O (0.1%; Sigma) and alcian blue (1%; Sigma). For immunostaining of collagen II and collagen I, the sections were incubated for 30 min in blocking buffer (1% BSA and 0.25% Triton X-100 in 0.9% saline) and then incubated with mouse anti-rabbit collagen II and I antibodies (1 : 100 dilution; Calbiochem) for 1 h at room temperature, respectively. Samples were then incubated with fluorescein isothiocyanate-conjugated goat anti-mouse IgG secondary antibody (1 : 200 dilution; Invitrogen) for 1 h at room temperature. Fluorescent images were acquired using the fluorescence microscope.

### 2.7. Quantitative Reverse Transcriptase-PCR (qRT-PCR)

To isolate total RNA, 15–20 cell-laden beads were harvested on days 0 and 35 and dissolved in 200 *μ*L of TRIzol reagent (Invitrogen) and RNA extraction was performed according to manufacturer's instruction. One microgram of total RNA was reverse transcribed into cDNA. qRT-PCR was performed using Brilliant III Ultra-Fast SYBR Green QPCR Master Mix (Agilent Technologies) on Mx300 real-time PCR system (Stratagene). Gene expression of collagen I, collagen II, and collagen X was analyzed. GAPDH was used as a housekeeping gene. For each gene of interest, relative expression level from day 35 to day 0 was calculated using ΔΔCt method. Each test was performed in triplicate.

### 2.8. Subcutaneous Implantation of Cell-Laden Alginate Beads in Nude Mice

After 35 d of dynamic culture, 20 cell-laden alginate beads from each culture were harvested and implanted into the back subcutis of nude mice to evaluate ectopic chondrogenesis. Mice were sacrificed at day 28  after implantation and implants were excised from host tissues and subjected to MTT assay, quantification of DNA and GAG contents, and qRT-PCR.

### 2.9. Statistical Analysis

Numerical data were represented as mean ± standard deviation. Difference between groups was evaluated by Student's *t*-test. Statistical significance was set at a *P* value of <0.05.

## 3. Results and Discussion

### 3.1. Experimental Design

The dedifferentiation of chondrocytes during 2D expansion is disadvantageous for their application in cartilage regeneration [5]. Instead, when primary chondrocytes are cultured in 3D (e.g., encapsulated in a hydrogel), the dedifferentiation can be attenuated [13, 15, 16]. Moreover, 3D culture of dedifferentiated chondrocytes is also able to induce the redifferentiation towards a hyaline phenotype [18, 19]. Alginate gel is extensively applied for 3D culture due to its simple and cytocompatible cell encapsulation process and has been shown to maintain the chondrocyte phenotype [15, 16, 19, 26]. On the other hand, mechanical stimulation is beneficial in the chondrogenesis [27]. Dynamic culture in spinner flasks has been suggested to be able to apply beneficial mechanical stimulation on chondrocytes [21, 22]. Therefore, in the present study, whether combining 3D and dynamic culture would be beneficial for maintaining the chondrocyte phenotype during* in vitro* culture was proposed.

**Figure 1:**
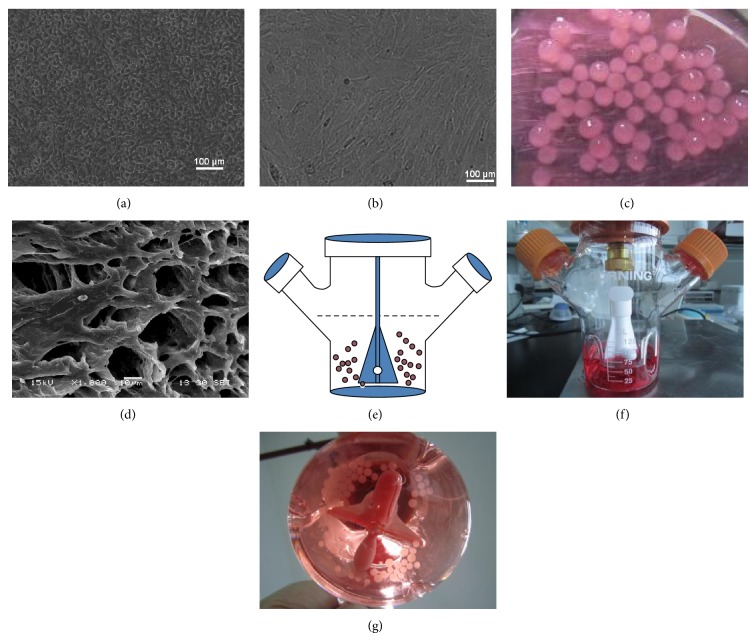
The setup of 3D dynamic culture. Morphology of P1 (a) and P4 (b) rACs cultured in 2D was observed under phase contrast microscope. (c) Gross view of cell-laden alginate beads; (d) SEM image of the interior microstructure of alginate beads laden with P1 rACs in chondrogenic medium for 28 d; (e) schematic illustration of suspension culture of cell-laden alginate beads in a spinner flask; (f) gross view of a spinner flask; (g) cell-laden alginate beads in a spinner flask.

To study the differentiation and redifferentiation of chondrocytes in this culture system, P1 and P4 rACs were applied, respectively. Chondrocytes of P1 are generally considered nondedifferentiated [28] and authentic chondrocyte dedifferentiation is often developed after 4 passages upon 2D expansion, after which it is considered that the redifferentiation cannot be achieved in 3D culture without chondrogenic induction factors [4, 29]. P4 rACs displayed a drastic morphological change from a rounded, cobble-like shape of P1 cells (Figure 1(a)) into the fibroblast-like appearance (Figure 1(b)), confirming the dedifferentiation of rACs after 4 passages [30].  Figure 1(c) showed the gross view of beads encapsulated with rACs having a diameter of 2-3 mm and an integral spherical morphology. The interior microstructure of cell-laden beads after dehydration under SEM was shown in Figure 1(d). As illustrated in Figures 1(e)–1(g), beads were loaded into a spinner flask and remained suspended under agitation of 50 rpm using a suspending magnetic stirring bar. With two different media (chondrocyte growth and chondrogenic media) and two different cells (P1 and P4 rACs), four different cultures were defined, that is, Ind-P1rAC, Con-P1rAC, Ind-P4rAC, and Con-P4rAC.

### 3.2. Viability and Growth of rACs in Alginate Beads

**Figure 2:**
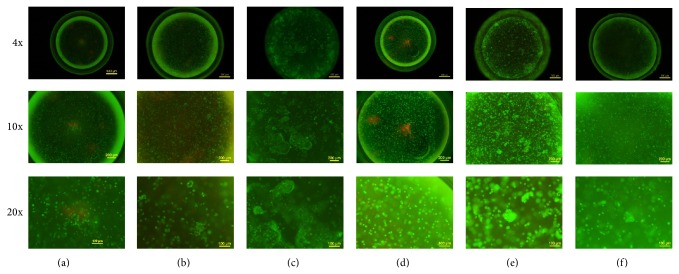
Live/dead staining of rACs in alginate beads. (a) P1 rACs on day 0; (b) P1 rACs in chondrogenic medium for 35 d (Ind-P1rAC); (c) P1 rACs in growth medium for 35 d (Con-P1rAC); (d) P4 rACs on day 0; (e) P4 rACs in chondrogenic medium for 35 d (Ind-P4rAC); (f) P4 rACs in growth medium for 35 d (Con-P4rAC). Fluorescent images were taken at 4x, 10x, and 20x magnifications. Green indicates live cells and red indicates dead cells.

After 35 d in culture, alginate beads retained the intact spherical morphology as on day 0 (Figure 2, magnification of 4x). Based on live/dead staining, both P1 and P4 cells uniformly dispersed as single cells, displaying a round morphology and only few cells died on day 0 (Figures 2(a) and 2(d)). On day 35, rACs remained round and viable in all cultures, except Ind-P1rAC showing slightly noticeable cell death (Figures 2(b), 2(c), 2(e), and 2(f)).

It was noted that on day 35, there emerged cellular aggregates in all cultures, which were most prominent for Con-P1rAC, followed by Ind-P4rAC (Figures 2(b), 2(c), 2(e), and 2(f)). Formation of aggregates had been reported previously when encapsulating chondrocytes in hydrogels and considered as a marker of chondrogenesis [15, 23, 31]. For P1 rACs, growth medium was more favorable for aggregate formation than chondrogenic medium (Figures 2(b) and 2(c)), suggesting that a chondrogenic medium might not be beneficial for* in vitro* culture of nondedifferentiated chondrocytes. For P4 cells, chondrogenic medium supported more efficient formation of aggregates (Figures 2(e) and 2(f)), indicating that a chondrogenic medium was favorable for redifferentiation of dedifferentiated chondrocytes in 3D dynamic condition. Interestingly, more aggregates were formed in Ind-P4rAC than Ind-P1rAC. These results demonstrated the distinct responses of P1 and P4 rACs to culture medium in 3D dynamic culture.

**Figure 3:**
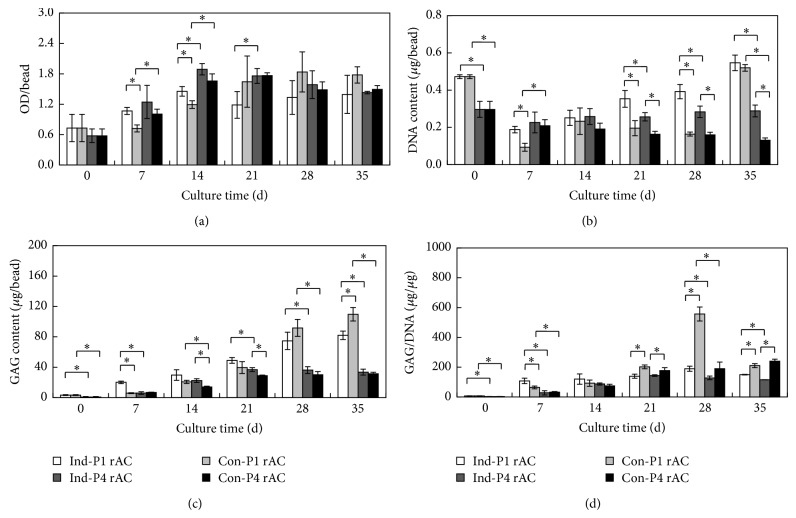
Growth and GAG production of rACs in alginate beads. (a) MTT assay; (b) DNA content; (c) GAG content; (d) GAG/DNA. Captions were defined as in Figure 2. Asterisk indicates *P* < 0.05.

Growth of rACs in alginate beads was assessed by MTT assay and DNA quantification. Based on MTT assay, the OD values initially increased with time and reached a plateau later for all four cultures (Figure 3(a)). This was consistent with a previous report, confirming that initially 2D cultured chondrocytes could quickly adapt to 3D condition [32]. In addition, while there were some differences at the beginning among four cultures, the OD values became similar after 28 d. For P1 rACs, OD values were slightly higher in chondrogenic medium than growth medium on both days 7 and 14. For P4 cells, there was no significant difference in the two media. Notably, OD values for P4 rACs were slightly greater than those of P1 rACs before day 21 in both media. As shown in Figure 3(b), DNA content showed a rather disparate trend compared to the OD values of MTT assay. On day 0, DNA content of P1 rACs was significantly higher than that of P4 rACs. For P1 rACs, DNA content decreased on day 7 in both media and then gradually increased till the end of culture, reaching a slightly higher level than that on day 0. This was slightly different from the finding by Almqvist et al., wherein a continuous increase in DNA content for primary chondrocytes encapsulated in alginate beads in static culture was observed [33]. It was presumed that such an acute decrease in DNA content on day 7 might be due to the potential mechanical perturbation enforced by fluid shear force in the spinner flask. For P4 rACs, DNA content varied in the two media. While DNA content gradually declined with time in growth medium, it remained relatively constant in chondrogenic medium. Studies have suggested that dedifferentiated chondrocytes might acquire mesenchymal stem cells-like properties, thus becoming anchorage-dependent and nonproliferative in alginate gel in growth medium [7, 34, 35]. However, upon the chondrogenic stimulation, dedifferentiated chondrocytes regained a chondrocytic phenotype, being able to thrive in 3D microenvironment [34, 35].

### 3.3. Cartilaginous ECM Production and Gene Expression of rACs in Alginate Beads

GAG production is a hall marker of chondrogenesis. As summarized in Figure 3(c), the gradual accumulation of GAG was observed in all cultures, suggesting that such a 3D dynamic culture supported* in vitro* chondrogenesis. However, P1 rACs deposited significantly a greater amount of GAG than P4 rACs, which might suggest that redifferentiated P4 cells still had an inferior chondrocytic phenotype than primary cells. Previous studies had reported a complete recovery of the chondrocytic phenotype for chondrocytes passaged for 2-3 times when cultured in the alginate gel [19, 31]. However, for cells beyond 4 passages, induction of redifferentiation required the presence of additional chondrogenic factors (e.g., TGF-*β*) in addition to 3D condition [18]. In addition, although GAG content of P1 rACs in chondrogenic medium was higher than that in growth medium before day 21, it was surpassed by the later one after day 28, further confirming that growth medium was favorable for P1 cells in 3D dynamic culture. For P4 rACs, GAG content was also slightly higher in chondrogenic medium at earlier time points and became similar at the later stage in the two media. This was in contrast with the study by Jonitz et al., wherein P3 human chondrocytes in alginate beads could deposit GAG in chondrogenic medium on day 35 in a static culture but not in growth medium [34]. However, when GAG content was normalized to DNA content, which reflects GAG productivity of a cell, it was found that at the later stage of culture both P1 and P4 rACs had higher GAG/DNA in growth medium than chondrogenic medium (Figure 3(d)). It was further noted that GAG/DNA for P4 cells became close to that for P1 cells in growth medium on day 35, suggesting a successful redifferentiation of dedifferentiated rACs towards a chondrogenic phenotype.

**Figure 4:**
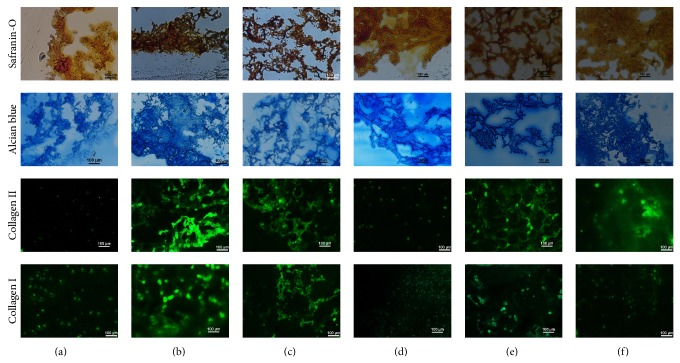
Histological analysis of rACs in alginate beads. (a) P1 rACs on day 0; (b) Ind-P1rAC on day 35; (c) Con-P1rAC on day 35; (d) P4 rACs on day 0; (e) Ind-P4rAC on day 35; (f) Con-P4rAC on day 35. Captions were defined as in Figure 2.

ECM production was further examined by histological staining. GAG was stained with Safranin-O and alcian blue dyes. As shown in Figure 4, albeit nonspecific staining of alginate by two dyes was present on day 0, and much stronger staining was discerned on day 35 for both P1 and P4 rACs. Consistent with GAG quantification, P1 rACs apparently produced more GAG than P4 cells. In addition, collagen II is the most abundant collagen type in hyaline cartilage and was detected using immunofluorescence staining. There was very little collagen II on day 0 for both cells (Figures 4(a) and 4(d)). After 35 d, Con-P1rAC showed the strongest positive staining among four cultures and for both cells, and collagen II deposition was greater in chondrogenic medium than in growth medium (Figures 4(b), 4(c), 4(e), and 4(f)). It has been implicated that mechanical forces such as fluid shear force in perfusion culture and dynamic compression might be unfavorable for the retention of ECM secreted by chondrocytes in engineered tissue constructs [36–38], which however was not noticed in the present study. Collagen I production was only detected for rACs in chondrogenic medium (Figures 4(b), 4(c), 4(e), and 4(f)). In addition, compared to collagen II, the increase in collagen I with culture time was trivial in all cultures, suggesting the differentiation towards a hyaline phenotype in this culture system [39].

**Figure 5:**
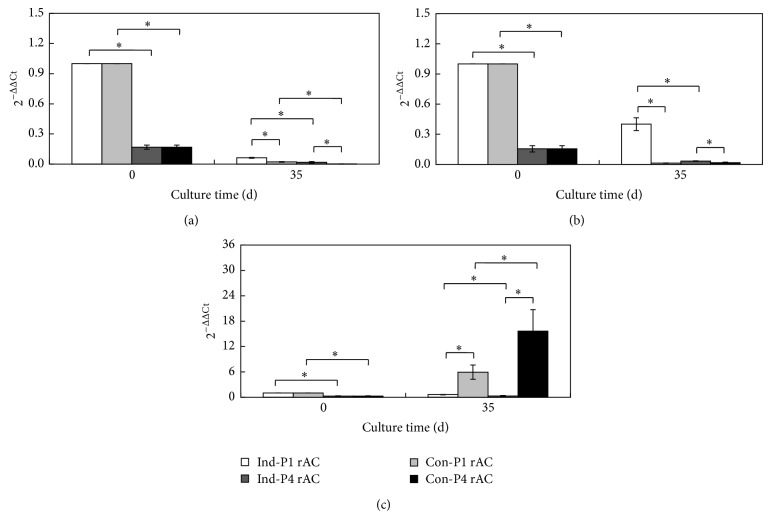
Gene expression of rACs in alginate beads. (a) Collagen type I; (b) collagen type II; and (c) collagen type X. Captions were defined as in Figure 2. Asterisk indicates *P* < 0.05.

Gene expression of collagens I, II, and X was analyzed using qRT-PCR (Figure 5). For all three genes, P4 rACs had lower expression levels than P1 rACs on day 0. Compared to that on day 0, gene expression of collagens I and II was downregulated in all four cultures on day 35. This was reasonable since cartilaginous gene expression generally peaks at early time points (between days 7 and 21) in response to chondrogenic stimulation [37, 40]. On day 35, P1 rACs showed higher expression of both collagens I and II than P4 rACs, except for collagen II in growth medium, which was consistent with a previous report of chondrocyte aggregates cultured in spinner flasks [22]. In addition, for both cells, expression of collagens I and II was higher in chondrogenic medium than that in growth medium and expression of collagen X was significantly suppressed in chondrogenic medium. Chondrogenic induction with TGF-*β* is often associated with the hypertrophy (i.e., upregulation of collagen X) [41]. It seemed that the 3D dynamic culture was able to attenuate this phenomenon in chondrogenic medium (i.e., with TGF-*β*1).

Chondrogenesis is initiated by cell proliferation, followed by cell aggregation, gene regulation, and deposition of characteristic ECM components [42]. It is worth noting that culture medium can be of great significance to* in vitro* chondrogenesis. Generally, serum-containing growth medium is preferred for cell division and serum-free medium is applied to stimulate chondrogenic differentiation [4]. Hence, at different stages of chondrogenesis, it may require different medium compositions to achieve the maximum chondrogenesis* in vitro*. In line with this, Zhang et al. found that a high content of serum in medium was unfavorable for chondrogenesis of rACs in alginate gel beads [32]. Recently, Wu et al. revealed that serum had significant effects on both production and spatial distribution of ECM by chondrocytes in 3D culture [43]. In the present study, for P1 rACs, while growth medium promoted cellular aggregation and GAG production, chondrogenic medium stimulated gene expression, and deposition of collagen II. For P4 cells, it became even more complicated since the survival of cells was closely linked to the differentiation process in alginate gel beads. It was found that while serum-free medium favored aggregate formation and cell proliferation as well as production of collagen II, GAG production was superior in growth medium for P4 rACs.

### 3.4. Ectopic Chondrogenesis in Nude Mice

**Figure 6:**
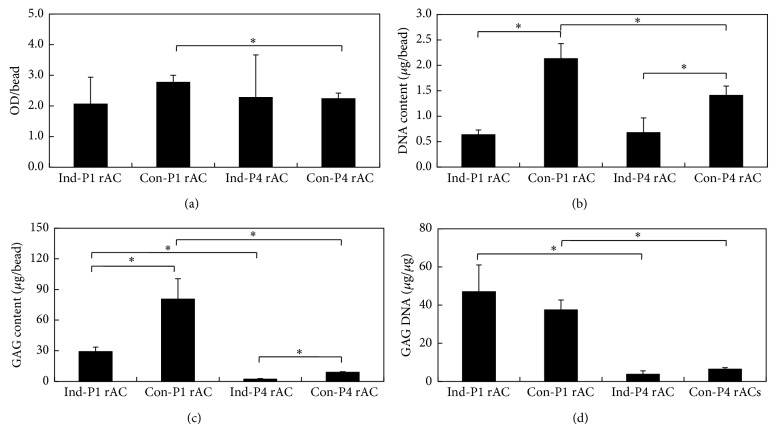
Cell growth and GAG production in implants. (a) MTT assay; (b) DNA content; (c) GAG/bead; (d) GAG/DNA. Asterisk indicates *P* < 0.05.

**Figure 7:**
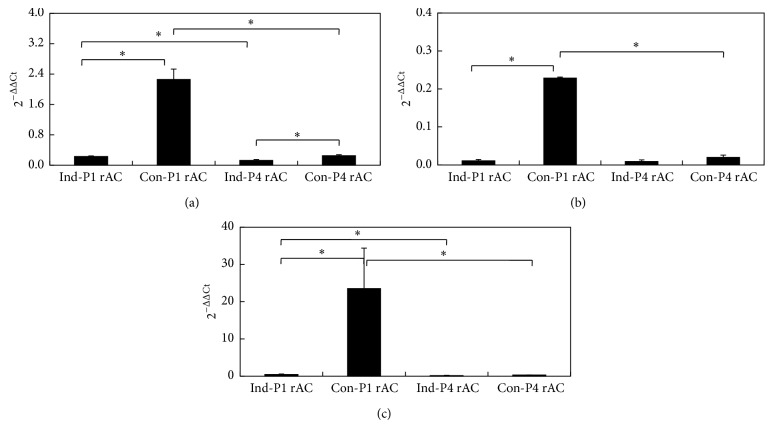
Gene expression of collagen type I (a), collagen type II (b), and collagen type X (c) of implants. Asterisk indicates *P* < 0.05.

Subcutaneous implantation in nude mice is an established model for studying the ectopic chondrogenesis [44]. For each culture, 20 beads were harvested and implanted. The contour of individual beads could be discerned beneath the skin immediately after implantation and the implants turned into integral blocks 28 d after implantation. MTT assay was performed to evaluate the growth of cells. The OD values remained at the similar levels in all implants, although those for Con-P1rAC were slightly higher than those for Con-P4rAC (Figure 6(a)). DNA content varied among implants (Figure 6(b)). For both P1 and P4 cells, DNA contents of implants from growth medium were higher. In addition, DNA content in Con-P1rAC was significantly higher than that in Con-P4rAC. In general, GAG contents of all implants were lower than those of respective beads prior to implantation (comparing the values in Figures 3(c) and 6(c)). GAG contents in implants from growth medium were significantly higher and implants of P1 cells secreted more GAG than those of P4 cells (Figure 6(c)). When GAG content was normalized to DNA content in implants, no significant difference was found between two media for both cells and GAG/DNA of P1 rACs was greater in both media (Figure 6(d)). Implants of Con-P1rAC had the highest gene expression of collagens I, II, and X (Figure 7). The expression of collagen II was similar for the implants of Ind-P1rAC, Ind-P4rAC, and Con-P4rAC (Figure 7(b)). For collagen I, implants from growth medium had higher expression for both cells and implants for P1 cells showed higher expression in both media (Figure 7(a)). For collagen X, implants from both media demonstrated higher expression for P1 cells (Figure 7(c)). Based on these results, P1 cells in growth medium demonstrated the greatest ectopic chondrogenesis, implicating that* in vivo* chondrogenesis is closely linked to* in vitro* culture conditions [44].

## 4. Conclusions

This study reported the effects of combining 3D encapsulation in alginate gel beads with spinner flasks for chondrocyte culture. It was demonstrated that 3D dynamic culture provided a suitable system supporting both differentiation and redifferentiation of chondrocytes. Notably, undedifferentiated chondrocytes (e.g., P1 cells) and dedifferentiated cells (e.g., P4 cells) displayed distinct behaviors in growth and differentiation. However, culture medium may also play critical roles in chondrocyte differentiation, which warrants further optimization to advance this 3D dynamic culture system. Moreover, these* in vitro* cultured chondrocytes-laden alginate gel beads might be directly implanted for cartilage tissue regeneration applications.
